# Reviewer acknowledgement 2014

**DOI:** 10.1186/s12866-015-0339-2

**Published:** 2015-02-04

**Authors:** Philippa K Harris

**Affiliations:** BioMed Central, 236 Gray’s Inn Road, London, WC1X 8HB UK

## Abstract

The editors of *BMC Microbiology* would like to thank all our reviewers who have contributed to the journal in Volume 14 (2014).

**Sadanandam Abbagani**

India

**Akio Abe**

Japan

**Abu-Bakr Abu-Median**

UK

**David Ackerley**

New Zealand

**Victoria Adetunji**

Nigeria

**Joe Aduse-Opoku**

UK

**Ameeta Agarwal**

USA

**Juhee Ahn**

South Korea

**Jose Ainsa**

Spain

**Olayinka Aiyegoro**

South Africa

**Per Åkesson**

Sweden

**Masato Akiba**

Japan

**Nashwan Al Naiemi**

Netherlands

**Munirul Alam**

Bangladesh

**Tiina Alamäe**

Estonia

**Sebastian Alberti**

Spain

**Pietro Alifano**

Italy

**Guenter Allmaier**

Austria

**Nerino Allocati**

Italy

**Ibrahim S Almssallem**

Saudi Arabia

**Juan Alonso**

Spain

**Francis Alonzo**

USA

**Hassanain Al-Talib**

Malaysia

**Christopher Alteri**

USA

**Adela Alvarez-Buylla**

Spain

**Sergio Alvarez-Perez**

Spain

**Artur Alves**

Portugal

**Ajith Anand**

UK

**Hany Anany**

Canada

**Bruno Andrade**

Brazil

**Antoine Andremont**

France

**Largus Angenent**

USA

**Fabrizio Anniballi**

Italy

**Richard Anthony**

Netherlands

**Josefa Anton**

Spain

**Patrícia Antunes**

Portugal

**Ibone Anza**

Spain

**Valério Aquino**

Brazil

**Victor H Aquino**

Brazil

**Alicia Aranaz**

Spain

**David Archer**

UK

**Carmen Ardanuy**

Spain

**Ronaldo C Argolo Filho**

Brazil

**Agustin Ariño**

Spain

**Stefania Arioli**

Italy

**Glen Armstrong**

Canada

**Dawn Arnold**

UK

**Daljit Singh Arora**

India

**Eva Arrebola**

Spain

**Kei Asai**

Japan

**Narito Asanuma**

Japan

**Gavin Ash**

Australia

**Diego Assis**

Brazil

**Stephen Aston**

Malawi

**Lea Atanasova**

Austria

**Matias Attene Ramos**

USA

**Graeme Attwood**

New Zealand

**Jonathon Audia**

USA

**Victoria Auerbuch**

USA

**Tiong Gim Aw**

USA

**Stéphane Aymerich**

France

**Parisa Badiee**

Iran

**Eduardo Bagagli**

Brazil

**Maria Bagattini**

Italy

**T Bagchi**

India

**Fabio Bagnoli**

Italy

**Linquan Bai**

China

**Bita Bakhshi**

Iran

**K Balakrishna**

India

**Ron Balczon**

USA

**Pablo Baldi**

Argentina

**David Baltrus**

USA

**Dennis Bamford**

Finland

**Silvia Yumi Bando**

Brazil

**Ehud Banin**

Israel

**Martina Barchitta**

Italy

**Sailen Barik**

USA

**Rodolphe Barrangou**

USA

**Matthieu Barret**

France

**Jennifer Barrila**

USA

**Hazel Barton**

USA

**Sylvette Bas**

Switzerland

**Marek Basler**

Switzerland

**Wagner Luiz Batista**

Brazil

**Andreas Baumler**

USA

**Ifor Beacham**

Australia

**Doerte Becher**

Germany

**Karsten Becker**

Germany

**Alexander Beliaev**

USA

**Boris Belitsky**

USA

**Christophe Michel Yves Beloin**

France

**Joseph Bender**

USA

**Celso Benedetti**

Brazil

**José Bengoechea**

UK

**Torbjörn Bengtsson**

Sweden

**Richard Bennett**

USA

**D. Michael Benson**

USA

**Zachary Bent**

USA

**Phillip Bergen**

Australia

**Jean-Marc Berjeaud**

France

**Michael Bester**

South Africa

**Rupak Bhadra**

India

**Ashima Bhardwaj**

India

**Fantahun Biadglegne**

Ethiopia

**Massimiliano Biagini**

Italy

**Piero A. Bianco**

Italy

**Michael Bidochka**

Canada

**Gursonika Binepal**

Canada

**Mina Bizic-Ionescu**

Germany

**Dominique Blanc**

Switzerland

**Matthias Bochtler**

Poland

**Levente Bodrossy**

Austria

**Patrick Boerlin**

Canada

**Stephane Bonacorsi**

France

**Bruno Bonaz**

France

**Dafne Bongiorno**

Italy

**Helena Boshoff**

USA

**Alex Bossers**

Netherlands

**Marco Bottino**

USA

**Francois Boudreau**

Canada

**Emeline Bouffartigues**

France

**Kostantinos (Kostas) Bourtzis**

Austria

**Guillaume Bouvet**

Canada

**Salvatore Bozzaro**

Italy

**Holger Brüggemann**

Denmark

**Fabio Braga**

Brazil

**Alessandra Bragonzi**

Italy

**Giovanni Brandi**

Italy

**Bernd Brandt**

Netherlands

**Catherine Branger**

France

**Alicia Bravo**

Spain

**Kelly Brayton**

USA

**Hans Breeuwer**

Netherlands

**James Brown**

USA

**Claude Bruand**

France

**Myriam Brugna**

France

**Jacob Bruin**

Netherlands

**Mark Brynildsen**

USA

**Maria Jose Buitrago**

Spain

**Alexander Bukreyev**

USA

**Irene Burckhardt**

Germany

**Gaelen Burke**

USA

**Lori L. Burrows**

Canada

**Vincent Burrus**

Canada

**Mary Burtnick**

USA

**Jeremy Burton**

Canada

**John Butcher**

UK

**Michael Calderwood**

USA

**Todd Callaway**

USA

**Angelika Callaway**

Germany

**Cinzia Calvio**

Italy

**Cesar Camilo**

Brazil

**Floriana Campanile**

Italy

**Ana Flávia Canovas Martinez**

Brazil

**Bin Cao**

Singapore

**Yongchang**

Cao China

**Rosa Capita**

Spain

**Franck Carbonero**

USA

**Etienne Carbonnelle**

France

**Silvia Cardona**

Canada

**Marta Carepo**

Portugal

**Paul Carlson**

USA

**Ian Carroll**

USA

**Katharine Carter**

UK

**Michelle Qiu Carter**

USA

**Cecilia Godoy Carvalhaes**

Brazil

**Irene Castano**

Mexico

**Luis Castillo**

Chile

**Angel Cataldi**

Argentina

**Nuno Cerca**

Portugal

**Fátima Cerqueira**

Portugal

**Chanhee Chae**

South Korea

**Stephane Chaillou**

France

**Jean Challacombe**

USA

**Neal Chamberlain**

USA

**Kok-Gan Chan**

Malaysia

**Sheela Chandra**

India

**Changxun Chang**

China

**Feng-Yee Chang**

Taiwan

**Wei-Lun Chang**

Taiwan

**Chien-Chung Chao**

USA

**Laetitia Chartrain**

UK

**Subhadeep Chatterjee**

India

**Pallab Chaudhuri**

India

**Susana Chavez-Bueno**

USA

**Chen Chen**

USA

**Guo-Qiang Chen**

China

**Shouwen Chen**

China

**Gongyou Chen**

China

**Hongxin Chen**

Finland

**Jiangang Chen**

USA

**Rachel Chen**

USA

**Si Chen**

China

**Xian-Ming Chen**

USA

**Lei Cheng**

China

**Luisa Cheng**

USA

**Yong-Hwa Cheong**

South Korea

**Chung Cheung**

Hong Kong

**Luciane Chimetto**

Brazil

**Bor-Sen Chiou**

USA

**Theodor Chitlaru**

Israel

**Yoon-E Choi**

South Korea

**Youngnim Choi**

South Korea

**Karuna Chourey**

USA

**Anuradha Chowdhary**

India

**Henry Choy**

USA

**Henrik Christensen**

Denmark

**Daniel K W Chu**

Hong Kong

**Duen-Yau Chuang**

Taiwan

**Patrick Cirino**

USA

**Rob Clubb**

USA

**Ahmet Yilmaz Coban**

Turkey

**Ronald Cobb**

USA

**Tom Coenye**

Belgium

**Arnaldo Lopes Colombo**

Brazil

**Bianca Colonna**

Italy

**Jan V Colpaert**

Belgium

**Elena Maria Comelli**

Canada

**Teresa Conneely**

USA

**Fernando L Consoli**

Brazil

**David Conway**

UK

**Daniel Cook**

USA

**Paul Cooper**

UK

**Peter Coote**

UK

**Victor Corman**

Germany

**Pierre Cornelis**

Belgium

**Antonio Correia**

Portugal

**Naima Gisela Cortes-Perez**

France

**Maria Costanzo**

USA

**Frederic Coulon**

UK

**Mathieu Coureuil**

France

**Melanie Courtot**

Canada

**Aurelie Crabbe**

USA

**Evan Crawford**

Canada

**Rebecca Creamer**

USA

**Tania Creczynski Pasa**

Brazil

**Ana Crespo Sempere**

Spain

**Tawni Crippen**

USA

**Oscar Cuevas**

Spain

**Scott Curry**

USA

**Patrick Curtis**

USA

**Sandra Da Re**

France

**Ana Lucia Darini**

UK

**Georges Daube**

Belgium

**Michael David**

USA

**Claudia D'Avila-Levy**

Brazil

**Robert Davis**

USA

**Bruna De Araujo Lima**

Brazil

**Christophe De Champs**

France

**Astrid De Greeff**

Netherlands

**Teresa De Kievit**

Canada

**Tania De Koning-Ward**

Australia

**Fernando De La Cruz**

Spain

**Clara De Los Reyes-Gavilan**

Spain

**Alejandra De Moreno De Leblanc**

Argentina

**Hilde De Reuse**

France

**Wanderley De Souza**

Brazil

**Annelies Declercq**

Belgium

**Margaret Deighton**

Australia

**Julien Delmas**

France

**Hendrik Cornelis Den Bakker**

USA

**Ye Deng**

China

**Xin Deng**

USA

**Jonathan Dennis**

Canada

**Ashok Desai**

India

**David Deshazer**

USA

**Ruud Deurenberg**

Netherlands

**Josef Deutscher**

France

**Kevin Devine**

Ireland

**Giovanni Di Bonaventura**

Italy

**Pier Paolo Di Nocera**

Italy

**Amanda Dias**

Brazil

**Wanderley Dias Da Silveira**

Brazil

**Alexander Dikiy**

Norway

**Mark Dillingham**

UK

**Yousong Ding**

USA

**Sylvatrie-Danne Dinzouna-Boutamba**

South Korea

**Alexander Dmitriev**

Russian Federation

**Ulrich Dobrindt**

Germany

**Harshavardhan Doddapaneni**

USA

**Delfina C. Dominguez**

USA

**Tao Dong**

Canada

**Osaana Donkor**

Australia

**Nicole Donofrio**

USA

**Timothy Douglas**

Belgium

**Lorenzo Drago**

Italy

**Michel Drancourt**

France

**Lorraine Draper**

Ireland

**Klaus Dreisewerd**

Germany

**Brian Driscoll**

Canada

**Mignon Du Plessis**

South Africa

**Kangmin Duan**

Canada

**Jean-Frederic Dubern Dubern**

UK

**Roger Dumke**

Germany

**Paul Dunlap**

USA

**Maya Dymova**

Russian Federation

**Garth Ehrlich**

USA

**Michael Ehrmann**

Germany

**Sigrun Eick**

Switzerland

**Mohamed Elasri**

USA

**Karin Elberse**

Netherlands

**Lindsay Eltis**

Canada

**Akihito Endo**

Finland

**Susanne Engelmann**

Germany

**Matt Erdman**

USA

**Joachim Ernst**

Germany

**Benjamin Evans**

UK

**Matthieu Eveillard**

France

**Dean Everett**

UK

**Joana Falcao Salles**

Netherlands

**Gang Fang**

USA

**Michael Federle**

USA

**Wen-Hai Feng**

China

**Francesco Feo**

Italy

**Chiara Ferrario**

Italy

**Ana Sofia Ferreira**

Portugal

**Ana Cristina Ferreira**

Portugal

**Susana Ferreira**

Portugal

**Antonio Ferreira-Pereira**

Brazil

**Paul Fidel**

USA

**Tomas Fiedler**

Germany

**Henri-Pierre Fierobe**

France

**Ersilia Fiscarelli**

Italy

**Klas Flardh**

Sweden

**Antje Flieger**

Germany

**Michael Flythe**

USA

**Anthony Fodor**

USA

**Jason Folster**

USA

**Anja Forche**

USA

**Christiane Forestier**

France

**Jane Foster**

Canada

**Timothy Foster**

Ireland

**George Fox**

USA

**Eelco Franz**

Netherlands

**Maria Fredriksson-Ahomaa**

Finland

**Stephen Free**

USA

**Shiri Freilich**

Israel

**Jason Fritzler**

USA

**Yong Fu**

USA

**John Fuerst**

Australia

**Kenji Fukuda**

Japan

**Irène Gabriel**

France

**Gabriela Gago**

Argentina

**Massimiliano Galdiero**

Italy

**Rodrigo Galhardo**

Brazil

**Antonia Gallo**

Italy

**Giorgio Gambino**

Italy

**Han Ming Gan**

Malaysia

**Yunn Hwen Gan**

Singapore

**Katarzyna Garbacz**

Poland

**Rozenn Gardan**

France

**Marcia Garnica**

Brazil

**Giuliano Garofolo**

Italy

**Daniel Garrido**

Chile

**Carlo Garzelli**

Italy

**Andrew Gates**

UK

**Patrice Gaurivaud**

France

**Gregory Gauthier**

USA

**Purushottam Gawande**

Canada

**Beilei Ge**

USA

**Otto Geiger**

Mexico

**Elke Genersch**

Germany

**J Georam**

China

**Herve Gerard**

USA

**Ralf Gerhard**

Germany

**Roman Gerlach**

Germany

**Giovanni Gherardi**

Italy

**Jean-Christophe Giard**

France

**Cara Gibson**

USA

**Mark Gijzen**

Canada

**Christophe Gilbert**

France

**Jason Gill**

USA

**Osnat Gillor**

Israel

**Osnat Gillor**

USA

**Philippe Glaser**

France

**Anthony Glenn**

USA

**Bernard Glick**

Canada

**Ralph Goethe**

Germany

**Dip Kumar Gogoi**

India

**Mark Gomelsky**

USA

**Gregório Gomez**

Brazil

**Oscar Gomez-Duarte**

USA

**Kiréopori Michel Gomgnimbou**

France

**Ursula Gonzales-Barron**

Portugal

**Jesús Gonzalo-Asensio**

Spain

**David Gordon**

Australia

**Boris Görke**

Austria

**Susan Gottesman**

USA

**Marcelo Gottschalk**

Canada

**Gustavo Bueno Gregoracci**

Brazil

**Ashleigh Griffin**

UK

**Joel Griffitts**

USA

**Gideon Grogan**

UK

**Elisabeth Grohmann**

Germany

**Rita Grosch**

Germany

**Alexandru Mihai Grumezescu**

Romania

**Thomas Guillard**

France

**Hayati Gunes**

Turkey

**Fei Guo**

China

**Ulvi Gursoy**

Finland

**Daniela Gwiazdowska**

Poland

**Carlton Gyles**

Canada

**Åsa Gylfe**

Sweden

**Abderrahman Hachani**

UK

**Ted Hackstadt**

USA

**Ferry Hagen**

Netherlands

**Eleni Hagi-Pavli**

UK

**Matthias Hahn**

Germany

**George Hajishengallis**

USA

**Anders Hakansson**

USA

**Tsuneo Hakoyama**

Japan

**Lindsay Hall**

UK

**Ruth Hall**

Australia

**Michael Hamblin**

USA

**Lennart Hammarström**

Sweden

**Brian Hammer**

USA

**Sven Hammerschmidt**

Germany

**Melanie Hamon**

France

**Abdul Hamood**

USA

**Scott Handley**

USA

**Anne-Marie Hansen**

USA

**Eric Hansen**

USA

**Ryan Hansen**

USA

**Harapan Harapan**

Indonesia

**Robert Doug Hardy**

USA

**Axel Hartke**

France

**Colin Harwood**

UK

**Valerie Harwood**

USA

**Claudia Hase**

USA

**Bill Hausdorff**

Belgium

**Finbarr Hayes**

UK

**Qiang He**

USA

**Yan He**

China

**Johannes Hegemann**

Germany

**John Helmann**

USA

**Niels Bohse Hendriksen**

Denmark

**Adriano Henriques**

Portugal

**Mariana Henriques**

Portugal

**Stefan Hertwig**

Germany

**Robert Hettich**

USA

**Marc Heyndrickx**

Belgium

**Nawal Hijjawi**

Jordan

**Thomas Hill**

USA

**Paul Himes**

USA

**Tiny Motlatso Hlokwe**

South Africa

**Chai-Ling Ho**

Malaysia

**Paulo Ho**

Brazil

**Ngo Hoa**

Viet Nam

**Mark Hodson**

Australia

**Ludwig Eduard Hoelzle**

Germany

**Monica Hofte**

Belgium

**Matthew Holden**

UK

**Nicola Holden**

UK

**Seok Jong Hong**

USA

**Yeonchul Hong**

South Korea

**Gerda Horneck**

Germany

**Mal Horsburgh**

UK

**Jacqui Horswell**

New Zealand

**Alexander Horswill**

USA

**Olga Horvat**

Serbia

**Jaroslav Hrabak**

Czech Republic

**Olga Hruskova-Heidingsfeldova**

Czech Republic

**Edmond Hsieh**

Taiwan

**Li-Nan Huang**

China

**Hairong Huang**

China

**Xinxiang Huang**

China

**Bernhard Hube**

Germany

**Vit Hubka**

Czech Republic

**Olivier Huck**

France

**Katalin Hudak**

Canada

**David Hunnicutt**

USA

**William J. (Jim) Hunter**

USA

**Susan Huse**

USA

**Embriette Hyde**

USA

**Alexander Idnurm**

Australia

**Aamer Ikram**

Pakistan

**Larisa Ikryannikova**

Russian Federation

**Iliyan Iliev**

USA

**Fumio Imazeki**

Japan

**Can Imirzalioglu**

Germany

**Simon Ip Cho**

Denmark

**Lourdes Isaac**

Brazil

**Renata Ivanek**

USA

**Hiroaki Iwaki**

Japan

**Masaaki Iwaki**

Japan

**Radoslaw Izdebski**

Poland

**Abdul Jabbar**

Australia

**Robert Jackson**

UK

**William Jackson**

USA

**Dan Jacobson**

South Africa

**George Jacoby**

USA

**Kang Jae Seung**

South Korea

**Kaïs Jamoussi**

Tunisia

**Peter Janssen**

New Zealand

**Jonathan Jantsch**

Germany

**Chelliah Jayabaskaran**

India

**Kee Ching Jeng**

Taiwan

**Paul Jensen**

USA

**Hai Jiang**

China

**Yongqiang Jiang**

China

**Zhao Jianmin**

China

**Elena Jimenez-Ruiz**

UK

**Mona Johannessen**

Norway

**Timothy Johnson**

USA

**Antonio Juárez**

Spain

**Sheryl Justice**

USA

**Aras Kadioglu**

UK

**Barbara Kahl**

Germany

**Ayse Kalkanci**

Turkey

**Chinnaperumal Kamaraj**

India

**Susan Kaminskyj**

Canada

**Tatsuo Kanda**

USA

**Edna Kaneshiro**

USA

**Jeffrey Kaplan**

USA

**Panagiotis Karanis**

Germany

**Gábor Kardos**

Hungary

**Petr Karlovsky**

Germany

**Des Kashyap**

USA

**Sophia Kathariou**

USA

**Sandeep Kathju**

USA

**Yusuke Kato**

Japan

**Michael Katze**

USA

**Daniel Kearns**

USA

**Corinna Kehrenberg**

Germany

**Rebecca Keller**

Germany

**David Kelly**

UK

**Jan Keltjens**

Netherlands

**Steve Kerrigan**

Ireland

**Ali Keshavarzian**

USA

**Krishna Mohan Ketha**

USA

**Timothy Kidd**

Australia

**Sebastian Kiewnick**

Switzerland

**John Kiiru**

Kenya

**Dong-Eun Kim**

South Korea

**Nobutada Kimura**

Japan

**James Kistler**

UK

**Sakae Kitada**

Japan

**Motomitsu Kitaoka**

Japan

**Morten Kjos**

Netherlands

**Eline Klaassens**

Australia

**Todd Klaenhammer**

USA

**Hideo Kobayashi**

Japan

**Robin Köck**

Germany

**Gerwald Koehler**

USA

**Thilo Köhler**

Switzerland

**Joanna Kolmas**

Poland

**Anne-Brit Kolsto**

Norway

**Jian Kong**

China

**Mirjam Kooistra-Smid**

Netherlands

**Jens Kreth**

USA

**Krister Kristensson**

Switzerland

**Anastasiya Krivoruchko**

Russia

**Bastiaan Krom**

Netherlands

**Victor Krylov**

Russia

**Joanna Kubler-Kielb**

USA

**Masae Kuboniwa**

Japan

**Indira Kudva**

USA

**Hannah Kuhn**

Germany

**Peter Kuhnert**

Switzerland

**Ed Kuijper**

Netherlands

**Purnima Kumar**

USA

**Surinder Kumar**

India

**Manikuntala Kundu**

India

**Souvik Kusari**

Germany

**Gabriel Kuty Everett**

USA

**Andrea Kwa**

Singapore

**Young Min Kwon**

USA

**Roberto La Ragione**

UK

**Jouni Laakso**

Finland

**Michael Lafleur**

USA

**Erh-Min Lai**

Taiwan

**Yi-Chyi Lai**

Taiwan

**Erwin Lamping**

New Zealand

**Chung-Yu Lan**

Taiwan

**Ellen Lanckacker**

Belgium

**Cornelia Landersdorfer**

Australia

**Frank Landgraf**

Germany

**Paolo Landini**

Italy

**Christopher Landowski**

Finland

**Sue Lang**

UK

**Jeroen Langereis**

Netherlands

**Martin Lappann**

Germany

**Jesper Larsen**

Denmark

**Emmanuelle Lauber**

France

**Mark Lawrence**

USA

**Alain Le Faou**

France

**Julie Ledford**

USA

**Isabelle Leduc**

USA

**Bok Luel Lee**

South Korea

**Dong-Yup Lee**

Singapore

**Jintae Lee**

South Korea

**Jungkwan Lee**

South Korea

**Lan-Ying Lee**

USA

**Dong-Woo Lee**

South Korea

**Hung-Chang Lee**

Taiwan

**Peter Lee**

Japan

**Sang-Suk Lee**

South Korea

**Michael Lehker**

USA

**Benfang Lei**

USA

**Julian Leibowitz**

USA

**James Leigh**

UK

**Tomasz Leski**

USA

**Olivier Lesouhaitier**

France

**Darren Letley**

UK

**Celine Levesque**

Canada

**Shawn Lewenza**

Canada

**Janina Lewis**

USA

**Huiying Li**

USA

**Fuli Li**

China

**Zhou Li**

USA

**Chunguang Liang**

Germany

**Lothar Lilge**

Canada

**Gipsi Lima Mendez**

Belgium

**Ching-Hsuan Lin**

Taiwan

**Xiaorong Lin**

USA

**Peter Lindblad**

Sweden

**Catharina Lindholm**

Sweden

**Karin Lindkvist-Petersson**

Sweden

**Steven Lindow**

USA

**Ruey-Fen Liou**

Taiwan

**George Liu**

USA

**Don Liu**

USA

**Zheng-Fei Liu**

China

**Shing-Hwa Liu**

Taiwan

**Xiufan Liu**

China

**Karen G Lloyd**

Denmark

**Jeffrey Locke**

USA

**Shawn Lockhart**

USA

**Bettina Löffler**

Germany

**Catherine Logue**

USA

**John Londesborough**

Finland

**Catia Longhi**

Italy

**Jose Lopez-Bucio**

Mexico

**Emilia López-Solanilla**

Spain

**Graciela Lorca**

USA

**Charles Lovell**

USA

**Chun Lu**

China

**Chengping Lu**

China

**Sangwei Lu**

USA

**Xiaonan Lu**

Canada

**Taina Lundell**

Finland

**Feng Luo**

USA

**Agnese Lupo**

Switzerland

**Andriy Luzhetskyy**

Germany

**David Mabey**

UK

**Donna Maccallum**

UK

**Robert Mach**

Austria

**Andres Mäe**

Estonia

**Veerle Maervoet**

Belgium

**Marcello Magri**

Brazil

**Eshwar Mahenthiralingam**

UK

**Rehab Mahmoud Abd El-Baky**

Egypt

**Jim Mahony**

Canada

**Tim Maisch**

Germany

**Aparna Maiti**

USA

**Kira Makarova**

USA

**Jose Bruno Malaquias**

Brazil

**Carolina Maldonado**

Argentina

**Pawan Malhotra**

India

**Caterina Mammina**

Italy

**Shannon Manning**

USA

**Ben Marais**

Australia

**Marina Marcet-Houben**

Spain

**Julian Marchesi**

UK

**Abelardo Margolles**

Spain

**Marilis Marques**

Brazil

**Robyn Marsh**

Australia

**Sara Martí**

France

**Rebeca Martin Rosique**

France

**Luis Martinez**

USA

**Maria Carmen Martinez Cuesta**

Spain

**Manuel Martinez Garcia**

Spain

**José Martinez-De-Oliveira**

Portugal

**Elisabete Martins**

Portugal

**Ellen Martinson**

USA

**Julio Massaharu Marubayashi**

Brazil

**Ramon Massana**

Spain

**Pedro A. Mateos Gomez**

USA

**John D Mathews**

Australia

**William Matias**

Brazil

**Kazunori Matsuda**

Japan

**Shigenobu Matsuzaki**

Japan

**Ann Matthysse**

USA

**Leena Maunula**

Finland

**Catherine May**

South Africa

**Luca Mazzon**

Italy

**Paul McAdam**

UK

**Jason McArthur**

Australia

**Anne McBride**

USA

**Christine McCartney**

UK

**John McCulloch**

Brazil

**Patrick McDermott**

USA

**Christopher McDevitt**

Australia

**Michael McShan**

USA

**Marcela Medina**

Argentina

**Samina Mehnaz**

Pakistan

**Marjolein Meijerink**

Netherlands

**Merit Melin**

Finland

**Melha Mellata**

USA

**Angela Melton-Celsa**

USA

**Tom Mendum**

UK

**Marco Meola**

Switzerland

**Elena Mercade**

Spain

**Muriel Mercier-Bonin**

France

**Scott Michael**

USA

**Ivan Mijakovic**

Sweden

**Katarina Mikusova**

Slovakia

**William Miller**

USA

**Kildare Miranda**

Brazil

**Oskar Modin**

Sweden

**Anne Moir**

UK

**James Moir**

UK

**Igor Mokrousov**

Russia

**Ian Molineux**

USA

**Vicente Monedero**

Spain

**John Moore**

South Africa

**Giulia Morace**

Italy

**Jacob Moran-Gilad**

Israel

**Cristiano Moreira**

Brazil

**Lilian Moreira**

Brazil

**Alison Morris**

USA

**Dimitris Mossialos**

Greece

**Jerome Mounier**

France

**David Moyes**

UK

**Kasey Moyes**

USA

**Pranab Mukherjee**

USA

**Suman Mukhopadhyay**

USA

**Thokur Sreepathy Murali**

India

**Mariano Jordi Muria Gonzalez**

Australia

**Ashok Naglot**

India

**Basavraj Nagoba**

India

**Shuichi Nakamura**

Japan

**Tsutomu Nakamura**

Japan

**Beiyan Nan**

USA

**Renu Nandakumar**

USA

**Vinay Nandicoori**

India

**Christian Napoli**

Italy

**Kalimuthusamy Natarajaseenivasan**

India

**William Navarre**

Canada

**Amadou Ndiaye**

Senegal

**Luis Nero**

Brazil

**Joseph Nesme**

France

**Elisabeth Neumann**

Brazil

**Danielle Neut**

Netherlands

**Irene Newton**

USA

**Dao Nguyen**

Canada

**Marisa F Nicolás**

Brazil

**Timo Niedermeyer**

Germany

**Kaare Magne Nielsen**

Norway

**Christina Nielsen-Leroux**

France

**Ludwig Niessen**

Germany

**Vladyslav Nikolayevskyy**

UK

**Mikeljon Nikolich**

USA

**Masayoshi Nishiyama**

Japan

**Clement Nizak**

France

**Philippe Noirot**

France

**Rashed Noor**

Bangladesh

**Mari Norgren**

Sweden

**Kazuto Nosaka**

Japan

**Angela Novais**

Portugal

**Alvaro Cantini Nunes**

Brazil

**Silvia Cristina Nunez**

Brazil

**Tuula Nyman**

Finland

**David O'Callaghan**

France

**Mary O'Connell Motherway**

Ireland

**Marco Rinaldo Oggioni**

UK

**Michael Oglesbee**

USA

**Abiodun Ogunniyi**

Australia

**Mitsuo Ogura**

Japan

**Elizabeth Ohneck**

USA

**Anil Ojha**

USA

**Shiro Okumura**

Japan

**Manuela Oliveira**

Portugal

**Richard Oliver**

Australia

**Stephen G Oliver**

UK

**Steven Olsen**

USA

**Madeleine Opitz**

Germany

**Timothy Opperman**

USA

**Joaquin Ortega**

Canada

**Yukio Oshiro**

Japan

**Radim Osicka**

Czech Republic

**Carlos Osorio**

Spain

**Jacobus Ossewaarde**

Netherlands

**George O'Toole**

USA

**Hong-Yu Ou**

China

**Soufian Ouchane**

France

**Kazuhisa Ouhara**

Japan

**Ernesto Oviedo-Orta**

Italy

**Rabindra Padhy**

India

**Hsiu-Hua Pai**

Taiwan

**Giuseppe Palladino**

USA

**Javier Palma-Guerrero**

Switzerland

**Jeffrey Palumbo**

USA

**Elzbieta Pamula**

Poland

**Rekha Panchal**

USA

**Seraphim Papanikolaou**

Greece

**Joseph Papaparaskevas**

Greece

**Kai Papenfort**

Germany

**Maria Gabriela Paraje**

Argentina

**Jean-Marie Parel**

USA

**Griffith Parks**

USA

**Claudio Passariello**

Italy

**Miroslav Patek**

Czech Republic

**Cheryl Patten**

Canada

**Luciana Paulino**

Brazil

**Giovanni Pavanello**

Italy

**Martin Pavelka**

USA

**Shelley Payne**

USA

**Andrew Pekosz**

USA

**Vladimir Pelicic**

UK

**Jose Penades**

UK

**Ines Pereira**

Portugal

**Jose Perez-Martin**

Spain

**David Perez-Pascual**

France

**Michael Perlin**

USA

**Mirjam Perner**

Germany

**Derek Persoh**

Germany

**Joseph Peters**

USA

**Isabelle Petropoulos**

France

**Davies Mubika Pfukenyi**

Zimbabwe

**Gregory Phillips**

USA

**Jiri Pikula**

Czech Republic

**Pascal Pineau**

France

**Minna Pirhonen**

Finland

**Laure Plener**

Germany

**Jason Pogue**

USA

**Monica Ponder**

USA

**Robert Poole**

UK

**Robert Pope**

USA

**Jean-Charles Portais**

France

**Michael Poulsen**

Denmark

**Spyros Pournaras**

Greece

**Mohammad Pourshafie**

Iran

**Narayan Prasad**

India

**Subbarama Prasad**

India

**Chet Price**

USA

**Luigi Principe**

Italy

**Kidsadagon Pringproa**

Thailand

**Robert Proctor**

USA

**Birgit Pruess**

USA

**Aneta Ptaszynska**

Poland

**Johannes Putze**

Germany

**Victor Pylro**

Brazil

**Di Qin**

China

**Paola Quatrini**

Italy

**Yok-Ai Que**

Switzerland

**Jos Raaijmakers**

Netherlands

**Tracy Raivio**

Canada

**Govindan Rajamohan**

India

**Mirjana Rajilic-Stojanovic**

Serbia

**Geeta Ram**

USA

**Thandavarayan Ramamurthy**

India

**Jorge Ramirez-Prado**

Mexico

**Cayo Ramos**

Spain

**Rommel Thiago Juca Ramos**

Brazil

**Giordano Rampioni**

Italy

**Brian Raphael**

USA

**Jean-Philippe Rasigade**

France

**Bala Rathinasabapathi**

USA

**Pallab Ray**

India

**Rosemary Redfield**

Canada

**Nicolas Refojo**

Argentina

**Guislaine Refrégier**

France

**Ian Reid**

Canada

**Joice Reis**

Brazil

**Dacheng Ren**

USA

**Maria Aparecida Resende**

Brazil

**Fredrik Resman**

Sweden

**Patricio Retamal**

Chile

**Sandra Reuter**

UK

**Hannah Reynolds**

USA

**Jon Marc Rhoads**

USA

**Ryan Rhodes**

USA

**Dayana Ribeiro**

Brazil

**Maria Ricci**

UK

**Peter Richard**

Finland

**Anthony Richardson**

USA

**Jonathan Richardson**

UK

**Christian Riedel**

Germany

**Alain Rince**

France

**Nicolas Rispail**

Spain

**Simon Rittmann**

Austria

**Rafael Rivilla**

Spain

**Syed Riyaz-Ul-Hassan**

India

**Carlos Robello**

Uruguay

**Lydia Robert**

France

**Brian Robertson**

UK

**Michael Robeson**

USA

**Roy Robins-Browne**

Australia

**Iraida E Robledo**

USA

**Charles Rock**

USA

**Annie Rodolakis**

France

**Nuri Rodriguez-Del Valle**

Puerto Rico

**Naiara Rodriguez-Ezpeleta**

Spain

**Alexander Rodriguez-Palacios**

USA

**Luiz Roesch**

Brazil

**Geraint Rogers**

UK

**Holger Rohde**

Germany

**Fernando Rojo**

Spain

**Julian Rood**

Australia

**Edvaldo Rosa**

Brazil

**Maddalena Rossi**

Italy

**Damien Roux**

France

**Gary Rowley**

UK

**Anjana Roy**

UK

**Songlin Ruan**

China

**Patricia Ruas-Madiedo**

Spain

**Edward Ruby**

USA

**Maja Rupnik**

Slovenia

**Etienne Ruppe**

France

**Robert Ryan**

UK

**Patrik Rydén**

Sweden

**Alexander Ryss**

Russia

**Marcia S.C. Melhem**

Italy

**Nurettin Sahin**

Turkey

**Vikram Saini**

USA

**Imran Sajid**

Pakistan

**John Samelis**

Greece

**Benedita Sampaio-Maia**

Portugal

**Susan Sanchez**

USA

**Peter Sander**

Switzerland

**Todd Sandrin**

USA

**Gunnar Sandstrom**

Sweden

**Maurizio Sanguinetti**

Italy

**Naseer Sangwan**

India

**Sílvio Santos**

Portugal

**R. Tedjo Sasmono**

Indonesia

**Isabel Saur**

Australia

**Jean-Michel Savoie**

France

**Gary Sawers**

Germany

**Anthony Schaeffer**

USA

**Isabelle Schalk**

France

**Birgit Scharf**

USA

**Herb Schellhorn**

Canada

**Bernhard Schink**

Germany

**Ingo Schmitz**

Germany

**Olaf Schneewind**

USA

**Tanja Schneider**

Germany

**Theo Schuurs**

Netherlands

**Herbert Schweizer**

USA

**Falk Schwendicke**

Germany

**Ismael Secundino**

Mexico

**Nicola Segata**

Italy

**Verena Seidl-Seiboth**

Austria

**Salome Seiffert**

Switzerland

**Masafumi Seki**

Japan

**Tsutomu Sekizaki**

Japan

**David Sela**

USA

**Jae-Gu Seo**

South Korea

**Young-Su Seo**

South Korea

**Andrea Serraino**

Italy

**Alain L. Servin**

France

**Puja Shahi**

USA

**Mohd Shahir Shamsir**

Malaysia

**Weixing Shan**

China

**Rohit Sharma**

USA

**Vijay Sharma**

USA

**Edward Shaw**

USA

**Lindsey Shaw**

USA

**Weifeng She**

USA

**Ann Sheehy**

USA

**Don Sheppard**

Canada

**Min Shi**

USA

**Michael Shiloh**

USA

**Makoto Shimosaka**

Japan

**Anna Shore**

Ireland

**Suja Shrestha**

Canada

**Deborah Siegele**

USA

**Philippe Silar**

France

**Wanderson Silva**

Brazil

**Sónia Silva**

Portugal

**Bruna Simioni**

Brazil

**Durg Singh**

India

**Anthony Sinskey**

USA

**Marcelo Sircili**

Brazil

**Anna Kaarina Sivonen**

Finland

**Anders Sjostedt**

Sweden

**Arne Skorping**

Norway

**David Skurnik**

USA

**Troy Skwor**

USA

**Jaroslaw Slawinski**

Poland

**Mark Smeltzer**

USA

**Hilde Smith**

Netherlands

**Leanne Smith**

UK

**Noel H. Smith**

UK

**E Snelders**

Netherlands

**Patricia Sobecky**

USA

**Mario Soberón**

Mexico

**Gloria Soberon-Chavez**

Mexico

**Christian Sohlenkamp**

Mexico

**Christophe Sola**

France

**Fuping Song**

China

**Joerg Soppa**

Germany

**Daniel Sordelli**

Argentina

**Uffe B. Skov Sorensen**

Denmark

**Marco Soriani**

Italy

**Antonio Sorlozano**

Spain

**Maximiliano Sortino**

Argentina

**Henning Sorum**

Norway

**Tanya Soule**

USA

**Richard Sparling**

Canada

**Fred Sparling**

USA

**Felipe Sperandio**

Brazil

**Silvana Spindola De Miranda**

Brazil

**Beny Spira**

Brazil

**Andrea Squartini**

Italy

**Padma Srikanth**

India

**V. Mohana Srinivasan**

India

**Graham Stafford**

UK

**Alistair Standish**

Australia

**Vilma Anitta Stanisich**

Australia

**Nicola Stanley-Wall**

UK

**Stefania Stefani**

Italy

**Irene Stefanini**

Italy

**Daniel Stein**

USA

**Joanne Stevens**

UK

**Roselynn Stevenson**

Canada

**Christopher Stewart**

UK

**Ellen Stobberingh**

Netherlands

**Daniel Stoebel**

USA

**David Stopar**

Slovenia

**Douglas Storey**

Canada

**Gisela Storz**

USA

**Eckhard Strauch**

Germany

**Gerald Stübiger**

Austria

**Joy Sturtevant**

USA

**David Summers**

UK

**Hongmin Sun**

USA

**Kim Sun-Ju**

South Korea

**Biao Suo**

China

**Petri Susi**

Finland

**Alexander Suvorofv**

Russia

**Taichi Suzuki**

USA

**Yasuhiro Suzuki**

USA

**Lovisa Svensson**

Sweden

**Eva Sverremark-Ekström**

Sweden

**Kelly Swanson**

USA

**Izabela Swiecicka**

Poland

**George A. Syrogiannopoulos**

Greece

**Morovat Taherikalani**

Iran

**Kapil Tahlan**

Canada

**Toshio Tanaka**

Japan

**Kam Tang**

UK

**Hongzhi Tang**

China

**Arnaud Tanguy**

France

**Jiahui Tao**

USA

**Rick Tarleton**

USA

**Theodoros Tasopoulos**

Greece

**Alessandra Tata**

Canada

**Arianna Tavanti**

Italy

**Aldo Tavares**

Brazil

**Valentina Taverniti**

Italy

**John Taylor**

UK

**Neung Teaumroong**

Thailand

**Stephen Techtmann**

USA

**Ligia Teixeira**

Qatar

**Paula Teixeira**

Portugal

**Neus Teixidó**

Spain

**Christoph Tellenbach**

Switzerland

**Thomas Templeton**

USA

**Lee-Jeng Teng**

Taiwan

**Takeaki Tezuka**

Japan

**Casey Theriot**

USA

**Francois Thiaucourt**

France

**Michael Thon**

Spain

**Line Thorsen**

Denmark

**Ulf Thrane**

Denmark

**Thomas Thurnheer**

Switzerland

**Chang Fu Tian**

China

**Chaoguang Tian**

China

**Collin Timm**

USA

**Andrew Timms**

UK

**Helena Tlaskalova-Hogenova**

Czech Republic

**Andrew Tomaras**

USA

**Inmaculada Tomas-Carmona**

Spain

**Hung Ton-That**

USA

**Istvan Toth**

Hungary

**Makrina Totsika**

Australia

**Patricia Totten**

UK

**Jean-Nicolas Tournier**

France

**Takahito Toyotome**

Japan

**Elizabeth Tran**

Australia

**Ousmane Traoré**

France

**Gena Tribble**

USA

**Apichai Tuanyok**

USA

**Paul Tudzynski**

Germany

**Paul Tulkens**

Belgium

**Somying Tumwasorn**

Thailand

**Williams Turpin**

Canada

**Francesca Turroni**

Italy

**Jaya Tyagi**

India

**Heather Tyler**

USA

**Milton Typas**

Greece

**Toshiki Uchiumi**

Japan

**Kenji Ueda**

Japan

**Atsuko Ueki**

Japan

**Cirano Ulhoa**

Brazil

**Preejith Vachali**

USA

**Mariadhas Valan Arasu**

South Korea

**Sylvia Valdezate**

Spain

**Chris Van Der Gast**

UK

**Wil Van Der Reijden**

Netherlands

**Menno Van Der Voort**

Netherlands

**Jan Van Der Wolf**

Netherlands

**Jakko Van Ingen**

Netherlands

**Willem Van Leeuwen**

Netherlands

**Philippe Van Lint**

Belgium

**Leo Van Overbeek**

Netherlands

**Mario Vaneechoutte**

Belgium

**Bartel Vanholme**

Belgium

**Eveline Vasconcelos**

Brazil

**Jose A. Vazquez**

Spain

**Beatriz Vazquez-De-Aldana**

Spain

**Mohanan Veettil**

USA

**Keerthi Venkataramanan**

USA

**Marco Ventura**

Italy

**Naresh Verma**

Australia

**Alane Beatriz Vermelho**

Brazil

**Ramesh Vetukuri**

Sweden

**Ana Carolina Vicente**

Brazil

**Stalinraj Victor**

Netherlands

**Angelica Vieira**

Brazil

**Richard Villemur**

Canada

**Julio Villena**

Argentina

**Boris Vinatzer**

USA

**Marie-Joelle Virolle**

France

**Miguel Viveiros**

Portugal

**Maria Guadalupe Vizoso Pinto**

Argentina

**Birgit Voigt**

Germany

**Uwe Völker**

Germany

**Carol Von Dohlen**

USA

**Ingemar Von Ossowski**

Finland

**Evangelia Voulgari**

Greece

**David Walker**

USA

**John Wallace**

UK

**Matthew Wand**

UK

**Fengping Wang**

China

**Guangyi Wang**

USA

**Jinglin Wang**

China

**Ruibai Wang**

China

**Yingfeng Wang**

USA

**Shihua Wang**

China

**Hao Wang**

USA

**Zhang Wang**

USA

**Alice Wattam**

USA

**Jean-Frédéric F. Weber**

Malaysia

**Paul Webster**

USA

**Grzegorz Wegrzyn**

Poland

**Hailei Wei**

USA

**Jaroslav Weiser**

Czech Republic

**Tim Welch**

USA

**Carol Wells**

USA

**Annette Wensing**

Germany

**Michael Wessels**

USA

**Thomas West**

USA

**Benita Westerlund-Wikström**

Finland

**Nathan Weyand**

USA

**Malcolm White**

UK

**Terence Whitehead**

USA

**Lothar H. Wieler**

Germany

**Stefan Wieschalka**

Denmark

**Linette Willemsen**

Netherlands

**Paul Wilmes**

Luxembourg

**Jody Winter**

UK

**Thomas Wittum**

USA

**Walter Wohanka**

Germany

**Ron Wohrle**

USA

**Daniel Wolter**

USA

**Dominic Wong**

USA

**Jon Woods**

USA

**Martin Woodward**

UK

**Karl Wooldridge**

UK

**Nancy Woychik**

USA

**Brendan Wren**

UK

**Ming-Shiang Wu**

Taiwan

**Yimou Wu**

China

**Qiaobin Xiao**

USA

**Zhizhong Xin**

China

**Xinping Xu**

USA

**Toshiharu Yakushi**

Japan

**Mamoru Yamada**

Japan

**Takuji Yamada**

Japan

**Shinji Yamasaki**

Japan

**Aixin Yan**

Hong Kong

**Qingyun Yan**

China

**Liang Yang**

Denmark

**Soo-Jin Yang**

USA

**Liang Yang**

Singapore

**Sizhong Yang**

China

**Yunfeng Yang**

China

**Tanya Yatsunenko**

USA

**Chew Chieng Yeo**

Malaysia

**Semih Yilmaz**

Turkey

**Deborah Yoder-Himes**

USA

**Carolyn Young**

USA

**Arnaldo Zaha**

Brazil

**Maria Mercedes Zambrano**

Colombia

**Oscar Zaragoza**

Spain

**Olympia Zarkotou**

Greece

**Raffaele Zarrilli**

Italy

**Egija Zaura**

Netherlands

**Ming Zeng**

China

**Cheng-Cai Zhang**

France

**Jingxin Zhang**

China

**Duoying Zhang**

China

**Haili Zhang**

USA

**Rui Zhang**

China

**Yuguang Zhang**

China

**Qijing Zhang**

USA

**Hui Zhang**

China

**Jianguo Zhang**

China

**Jianzhong Zhang**

China

**Sen Zhang**

China

**Wei Zhang**

USA

**Zhaojie Zhang**

USA

**Jindong Zhao**

China

**Jinlei Zhao**

USA

**Li-Xing Zhao**

China

**Han Zheng**

China

**Yong Zhou**

USA

**Wei-Yun Zhu**

China

**James Zlosnik**

Canada

**Zhiyong Zong**

China

**Angeles Zorreguieta**

Argentina

**Huasong Zou**

China

**Mohammad Zubair**

India

**Sonia Zuñiga**

Spain

